# Acute high-intensity interval rowing increases thrombin generation in healthy men

**DOI:** 10.1007/s00421-016-3370-6

**Published:** 2016-04-12

**Authors:** Matthew J. Sedgwick, Matthew Thompson, Jack Garnham, Alice E. Thackray, Laura A. Barrett, Matthew Powis, David J. Stensel

**Affiliations:** School of Sport, Exercise and Health Sciences, Loughborough University, Loughborough, Leicestershire LE11 3TU UK; Department of Sport, Health and Nutrition, Leeds Trinity University, Leeds, UK; Pathway Diagnostics Ltd, Surrey, UK

**Keywords:** Blood coagulation, Cardiovascular disease risk, Exercise intensity, Triacylglycerol

## Abstract

**Purpose:**

High-intensity exercise induces several health benefits, but may acutely and transiently increase the risk of cardiovascular events due to thrombotic changes promoting blood coagulation and thrombin formation. This study examined the effects of high-intensity exercise on plasma thrombin generation and triacylglycerol concentrations.

**Methods:**

Sixteen healthy men completed two, 2-day conditions separated by 1 week. On day 1, participants rested (control) or completed four, 3-min high-intensity rowing intervals at an average rating of perceived exertion of 17 (exercise). Venous blood samples were collected pre- and post-intervention to determine plasma thrombin generation. On day 2, participants rested and consumed a glucose load (0 h) and high-fat meal (2 h). Fifteen venous blood samples were collected between 0 and 8 h to measure plasma thrombin generation and triacylglycerol concentrations.

**Results:**

On day 1, lag time was shorter and peak thrombin and endogenous thrombin potential were greater in the exercise than control condition (ES ≥ 0.37, main effect condition *P* ≤ 0.03), and post-intervention compared with pre-intervention (ES ≥ 0.49, main effect time *P* ≤ 0.003). The magnitude of the post-intervention change was greater in the exercise than control condition for all thrombin generation parameters (condition by time interaction *P* ≤ 0.05). On day 2, no differences in postprandial thrombin generation parameters were seen between conditions (*P* ≥ 0.21). The total area under the curve for triacylglycerol was lower in the exercise than control condition (ES = 0.34, *P* = 0.02).

**Conclusion:**

An acute bout of high-intensity interval rowing increased plasma thrombin generation immediately after exercise, but these differences were eliminated 16–24 h after exercise.

## Introduction

Blood haemostasis represents a complex interaction between platelets, coagulation and fibrinolysis to maintain the integrity of the vascular wall. An imbalance of this complex regulated system promotes an increased risk of clinical complications from atherosclerotic cardiovascular disease (Borissoff et al. 2011), which remains the leading cause of mortality and morbidity in Western society (World Health Organisation 2011). The coagulation system represents an important protagonist in the development of atherogenesis and atherothrombosis (Borissoff et al. 2011). Thrombin is a pivotal enzyme in the coagulation cascade catalysing the conversion of soluble fibrinogen to insoluble fibrin to accelerate clot formation (Crawley et al. 2007). Therefore, lifestyle interventions that improve the coagulation profile by targeting thrombin generation may lower the risk of atherosclerosis and promote important metabolic health benefits.

Endurance training studies highlight the potency of regular exercise to improve markers of coagulation status (Gram et al. 2015; Hilberg et al. 2013). Recent studies demonstrate that low-volume, high-intensity interval exercise elicits a myriad of health benefits that are comparable or even superior to those induced by higher volume, continuous aerobic exercise (Currie et al. 2013; Hood et al. 2011; Little et al. 2011; Trombold et al. 2013). However, acute and strenuous physical exertion may transiently increase the risk of thrombotic events and sudden cardiac death during and immediately after exercise (Thompson et al. 2007). Acute exercise provokes a transient hypercoaguable state (Posthuma et al. 2015), evidenced most consistently by global tests demonstrating a shortening of activated partial thromboplastin time (aPTT) (Gunga et al. 2002; Hilberg et al. 2002, 2003b; Menzel and Hilberg 2011). Furthermore, increases in surrogate markers of thrombin generation such as prothrombin activation fragment 1 + 2 (F1 + 2) and thrombin-antithrombin complex (TAT) have been supported (Hilberg et al. 2003a; Weiss et al. 2002), although this effect may be dependent on the exercise intensity (Cadroy et al. 2002; Menzel and Hilberg 2011; Weiss et al. 1998). The development of thrombin generation assays have made it possible to provide a global assessment of plasma thrombin generation over time, allowing the process of thrombin generation and coagulation potential to be captured with greater sensitivity (Hemker et al. 2003). However, we are not aware of studies that have examined the immediate effect of high-intensity exercise on thrombin generation using this novel technique, highlighting an important gap in our understanding which may have significant implications in determining the safety of high-intensity exercise.

The majority of waking hours are postprandial and, although many postprandial changes are transient in nature, the metabolic disturbances after a meal are unlikely to subside before subsequent meal consumption. Although the activation of coagulation parameters such as factor VII coagulant activity have been supported during postprandial lipaemia (Gill et al. 2001; Silva et al. 2003), evidence of increased postprandial thrombin generation has not been reported yet. Nevertheless, coagulation factor VII appears to be positively associated with postprandial triacylglycerol (TAG) concentrations (Silva et al. 2003), an established independent risk factor for cardiovascular disease (Nordestgaard et al. 2007), highlighting a potential procoagulant tendency in the postprandial period. Several recent studies highlight the potential efficacy of acute intermittent high-intensity exercise performed up to 18 h before a standardised meal to reduce postprandial TAG concentrations (Freese et al. 2011; Gabriel et al. 2012; Trombold et al. 2013). However, the effect of high-intensity exercise on postprandial thrombin generation the day after exercise is not known.

Therefore, the primary aim of this study was to examine acute changes in plasma thrombin generation immediately after a single session of high-intensity interval rowing exercise using a novel thrombin generation assay in healthy, recreationally active men. In addition, the effect of acute high-intensity interval exercise on postprandial thrombin generation and TAG, glucose and insulin concentrations was determined 16–24 h after exercise.

## Methods

### Participants

Following approval from the University Ethical Advisory Committee, 16 men aged 19.8–30.2 years were recruited and provided their written informed consent to participate in this study. Participants were non-smokers, not taking medication or dietary supplements and had no known history of cardiovascular disease or coagulation disorders.

### Anthropometry

Stature was measured to the nearest 0.01 m using a fixed stadiometer (Seca 222, Seca Ltd, Hamburg, Germany), and body mass was quantified to the nearest 0.1 kg using a digital scale (Adam CFW-150, Milton Keynes, UK). Body mass index was calculated as body mass (kg) divided by stature (m) squared. Waist circumference was determined as the narrowest point of the torso between the xiphoid process and the iliac crest. Triceps, biceps, subscapular and suprailiac skinfold thickness was measured to the nearest 0.2 mm on the right hand side of the body using skinfold callipers (Baty International, West Sussex, UK) to estimate body density (Durnin and Womersley 1974), and body fat percentage was calculated (Siri 1956).

### Preliminary exercise measurements

Following familiarisation with the stationary rowing ergometer (Model D, Concept II, Nottingham, UK), participants completed a 3 min warm-up, followed immediately by four, 3 min high-intensity rowing intervals. Heart rate was monitored continuously via short-range telemetry (Polar FS20, Polar Electro, Kempele, Finland), and the participant’s rating of perceived exertion (RPE) was recorded at the end of each interval using Borg’s 6–20 scale (Borg 1973). Participants were instructed to exercise at an intensity equivalent to 10 on the RPE scale during the warm-up (between ‘very light’ and ‘fairly light’), and the remaining intervals corresponded to a self-regulated RPE of 17 (‘very hard’).

### Experimental design

**Fig. 1:**
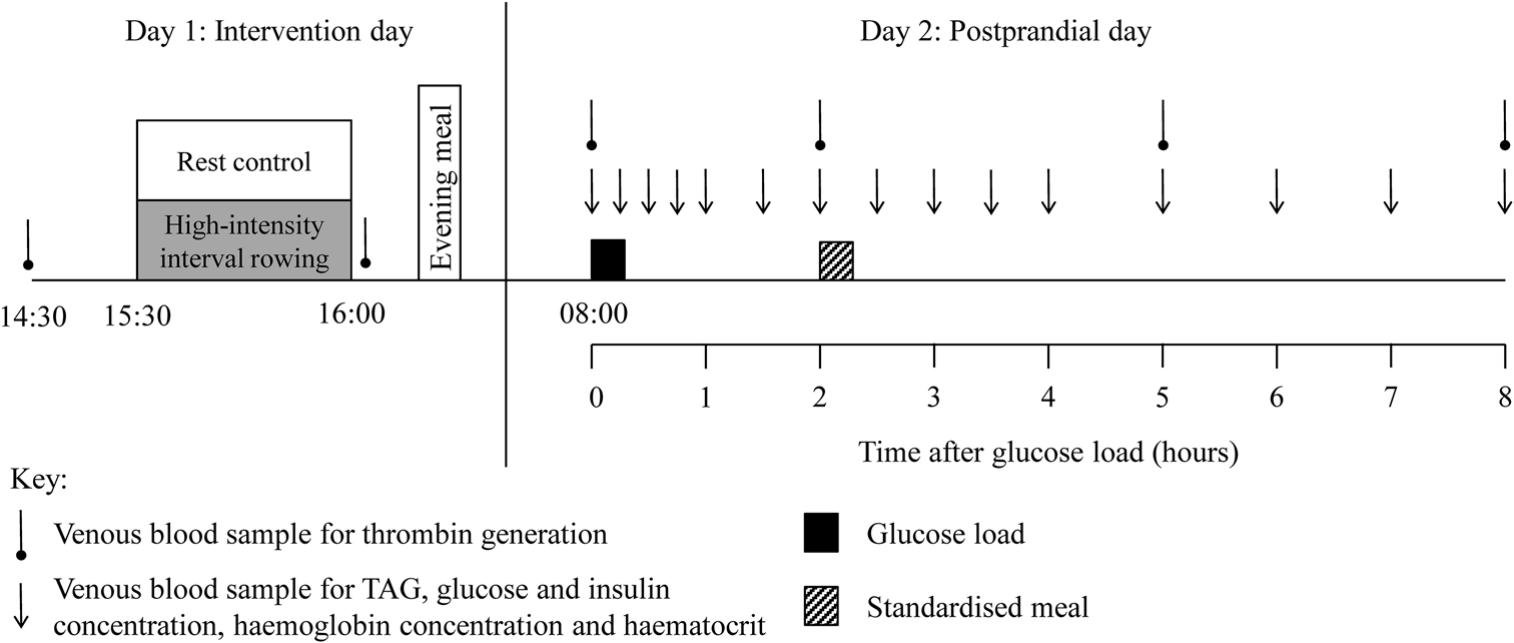
Diagram of the 2-day study protocol. TAG, triacylglycerol

Participants completed two, 2-day conditions (control and exercise) in a within-measures, counterbalanced, crossover design. The conditions were separated by at least 1 week and the study design is presented schematically in Fig. 1. Participants were asked to refrain from strenuous physical activity and not to consume alcohol or caffeine during the 48 h period before day 2 of each condition. They also completed a weighed dietary record during the same period of the first condition and replicated this diet before the subsequent condition.

### Day 1: intervention day

Participants arrived at the laboratory at 14:15 following a 2–3 h fast and a venous blood sample was taken after 15 min rest for the measurement of plasma thrombin generation. During the exercise session, participants completed a 3 min warm-up at a self-selected RPE of 10, followed immediately by four, 3 min rowing intervals at a self-selected RPE of 17 interspersed with 3 min passive recovery. Heart rate was monitored continuously and RPE was recorded at the end of each interval as described previously. Expired air samples were collected during the final minute of intervals 2 and 4 into Douglas bags (Cranlea and Company, Birmingham, UK). Oxygen uptake ($\dot{V}$O_2_) and carbon dioxide production were analysed using a paramagnetic oxygen analyser and an infrared carbon dioxide analyser (Servomex 1440, East Sussex, UK) and the volume of expired air was quantified using a dry gas meter (Harvard Apparatus Ltd, Kent, UK). Immediately after the cessation of exercise a further venous blood sample was collected. During the control condition, participants rested in the laboratory and a venous blood sample was taken at equivalent time points to the exercise condition.

### Day 2: postprandial day

Participants reported to the laboratory at 07:45 following a 10 h overnight fast and a fasting venous blood sample was taken after 15 min rest to determine plasma thrombin generation and TAG, glucose and insulin concentrations. They then consumed a 75 g glucose load (82.5 g dextrose monohydrate) in 300 ml water for breakfast, marking the start of the postprandial period. This breakfast was selected to represent the carbohydrate-rich breakfasts that are typically consumed in Westernised countries (Reeves et al. 2013), and to investigate the effect of high-intensity interval rowing on markers of insulin resistance and sensitivity. Subsequent venous blood samples were collected at 0.25, 0.5, 0.75, 1, 1.5, 2, 2.5, 3, 3.5, 4, 5, 6, 7 and 8 h after the ingestion of the glucose load. Plasma thrombin generation parameters were measured from samples collected at 0, 2, 5 and 8 h. Concentrations of TAG and glucose were measured from all samples, and insulin was measured from all samples except at 5 and 7 h. Participants consumed a standardised meal within 20 min immediately after the venous blood sample at 2 h. The meal consisted of white bread, butter, cheese, potato crisps, whole milk and chocolate milkshake powder, and provided 0.7 g fat (51 % of total meal energy), 1.2 g carbohydrate (37 %), 0.4 g protein (12 %) and 55 kJ energy per kilogram body mass. The time taken to consume the meal during the first condition was replicated in the remaining condition. Participants consumed water ad libitum between 2 and 8 h during the first condition; the ingested volume was replicated in the subsequent condition.

### Analytical methods

Venous blood samples were collected via venepuncture from an antecubital vein on day 1, and on day 2, using a cannula (Venflon, Becton–Dickinson, Helsingborg, Sweden) inserted into an antecubital vein. All samples were collected in the semi-supine position. For samples collected using a cannula, patency was maintained by flushing with non-heparinised saline [0.9 % (w/v) sodium chloride; Baxter Healthcare Ltd, Norfolk, UK]. Residual saline was discarded using a 2 mL syringe prior to sample collection. Samples were drawn into pre-chilled 5 mL citrate theophylline adenosine dipyridamole (CTAD) monovettes (Sarstedt, Leicester, UK) for the assessment of plasma thrombin generation on day 1 and day 2. To prevent the degradation of clotting factors, nine parts venous blood was added to one part of 0.106 M trisodium citrate (equivalent to 3.2 % trisodium citrate). Monovettes were immediately centrifuged at 1500×*g* for 10 min at 4 °C (Heraeus Labofuge 400R, Thermo Electron, Osterode, Germany). The top 2 mL of plasma supernatant was evenly aliquoted into two Eppendorf tubes (Eppendorf, Hamburg, Germany) and re-centrifuged under the same conditions. Subsequently, the top 500 µL of plasma supernatant from each tube was dispensed into Eppendorf tubes to obtain platelet-poor plasma, and samples were stored at −20 °C for subsequent analysis. Thrombin generation was measured in duplicate using a commercially available fluorogenic assay kit (Technothrombin^®^ TGA, Technoclone, Vienna, Austria) on a fully automated coagulation analyser (Ceveron^®^ Alpha, Technoclone, Vienna, Austria). The thrombin generation vs. time curve was established and the following parameters of thrombin activity were recorded: (1) lag time: time until thrombin burst; (2) peak thrombin: maximal concentration of thrombin formed; (3) endogenous thrombin potential: total enzymatic work performed by thrombin equated as the area under the thrombin generation curve. The inter- and intra-assay coefficient of variation was 7.7 and 4.5 %, respectively.

To determine the concentration of TAG, glucose and insulin on day 2, venous blood was collected into pre-chilled 9 mL EDTA monovettes (Sarsedt, Leicester, UK). Monovettes were centrifuged immediately at 1500×*g* for 10 min at 4 °C (Heraeus Labofuge 400R, Thermo Electron, Osterode, Germany). The plasma supernatant was aliquoted into Eppendorf tubes prior to storage at −20 °C for subsequent analysis. Plasma TAG and glucose concentration was analysed by enzymatic, colorimetric methods using a benchtop analyser (Pentra 400, HORIBA ABX Diagnostics, Montpellier, France). Plasma insulin concentration was analysed using a commercially available enzyme-linked immunoassay (Mercodia Insulin ELISA, Mercodia AB, Uppsala, Sweden). The within-batch coefficient of variation for TAG, glucose and insulin concentration was 1.7 % (1.20 mmol L^−1^), 0.5 % (7.19 mmol L^−1^) and 4.7 % (186.5 pmol L^−1^), respectively.

At each blood sampling point, haemoglobin concentration and haematocrit were quantified in duplicate to estimate acute changes in plasma volume (Dill and Costill 1974). Haemoglobin concentration was assessed using the cyanmethemoglobin method; 20 µL whole blood was added to 5 mL Drabkin’s solution and the absorbance was quantified photometrically at a wavelength of 546 nm (Cecil CE1011, Cecil Instruments, Cambridge, UK). Haematocrit was quantified using a microhaemoatocrit centrifuge and reader (Haematospoin 1300 Microcentrifuge, Hawksley and Sons Ltd, Sussex, UK).

### Statistical analysis

Data were analysed using the IBM SPSS Statistics software for Windows version 21.0 (IBM Corporation, New York, USA). Descriptive statistics illustrating the physical and physiological characteristics and exercise responses were calculated. The trapezium rule was used to calculate time-averaged total area under the curve (TAUC) values. The homeostasis model assessment of insulin resistance (HOMA-IR) (Matthews et al. 1985) and insulin sensitivity index (Matsuda and DeFronzo 1999) were calculated. Normality of the data was checked using Shapiro–Wilk tests. Normally distributed data are presented as mean (SD). Thrombin generation parameters, and TAG, glucose and insulin concentrations were not normally distributed and were natural log transformed prior to analysis. These data are presented as geometric mean (95 % confidence interval) and analysis is based on ratios of the geometric means and 95 % confidence intervals for ratios.

Linear mixed models repeated for condition (control and exercise) and time (pre- and post-intervention) were used to examine differences in thrombin generation parameters on day 1. Dietary intake, markers of insulin resistance and sensitivity, fasting concentrations and TAUC responses on day 2 were analysed using linear mixed models with condition (control and exercise) as the sole factor. Differences in thrombin generation parameters and TAG, glucose and insulin concentrations over the total 8 h postprandial period were examined using linear mixed models repeated for condition (control and exercise) and time. All linear mixed models included a random effect for each participant.

Statistical significance was accepted as *P* < 0.05 and absolute standardised effect sizes (ES) are included to supplement important findings. In the absence of a clinical anchor, an ES of 0.2 was considered the minimum important difference in all outcome measures, 0.5 moderate and 0.8 large (Cohen 1988). Graphical representations of results are presented as mean (SEM) to avoid distortion of the graphs.

## Results

### Participant characteristics

The physical characteristics of participants were as follows: age 23.4 (2.9) years, body mass 80.9 (13.9) kg, body mass index 24.7 (3.6) kg m^−2^, body fat 18.5 (4.2) %, waist circumference 84.6 (10.1) cm.

### Dietary intake

Average energy intake was similar during the 48 h before day 2 of the control and exercise conditions [10.1 (2.9) vs. 10.3 (3.3) MJ day^−1^, respectively; main effect condition *P* = 0.38]. Average 2-day macronutrient intake did not differ between the control and exercise conditions for protein [106 (26) vs. 113 (34) g day^−1^; main effect condition *P* = 0.22], carbohydrate [299 (116) vs. 302 (119) g day^−1^; main effect condition *P* = 0.67] or fat [94 (39) vs. 96 (44) g day^−1^; main effect condition *P* = 0.62], respectively.

### Rowing exercise responses

Participants completed the four high-intensity rowing bouts at a mean power output of 224 (49) W, and covered a mean distance of 772 (56) m per interval [3087 (224) m in total]. Mean $\dot{V}$O_2_ during the exercise session was 3.36 (0.76) L min^−1^, corresponding to 42 (9) mL kg^−1^ min^−1^, and the average respiratory exchange ratio was 1.05 (0.09). Mean heart rate was 179 (10) beats min^−1^, which represented 91 (5) % of age predicted maximum heart rate, and the average RPE was 17 (0) (‘very hard’ on the scale).

### Day 1: thrombin generation parameters pre- and post-intervention

**Fig. 2:**
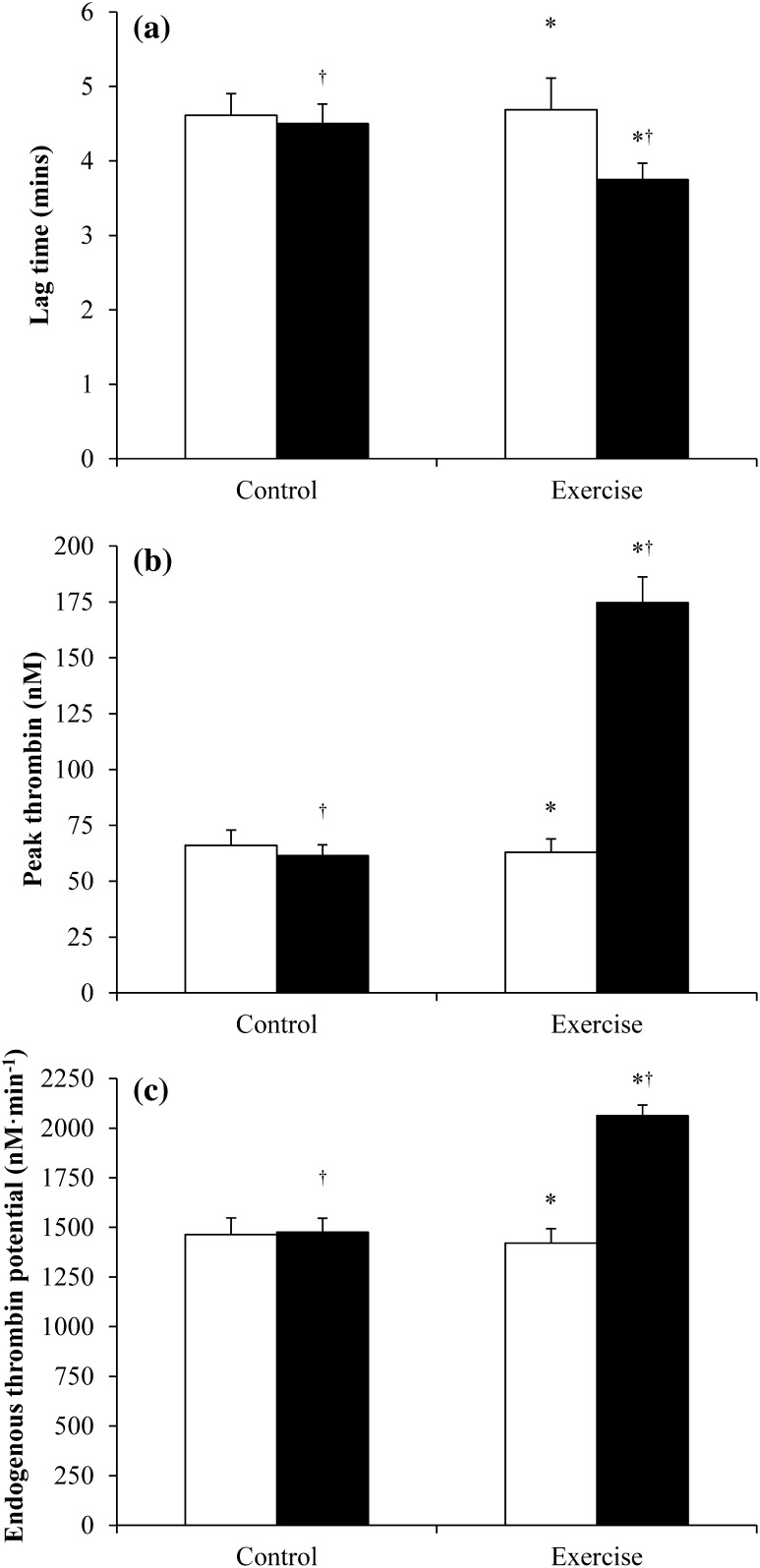
Plasma thrombin generation parameters measured pre- (*open square*) and post- (*closed square*) intervention in the control and exercise conditions (*n* = 16): **a** lag time; **b** peak thrombin; **c** endogenous thrombin potential. Values are mean (SEM). *Significant difference between exercise and control conditions, main effect condition *P* ≤ 0.03; ^†^significant difference between pre- and post-intervention, main effect time *P* ≤ 0.003

Plasma thrombin generation parameters measured pre- and post-intervention in the control and exercise conditions are displayed in Fig. 2. Lag time was 8 % lower in the exercise compared with the control condition (−15 to −1 %, ES = 0.37, main effect condition *P* = 0.03), and 11 % lower post-intervention compared with pre-intervention (−17 to −4 %, ES = 0.49, main effect time *P* = 0.003). The magnitude of reduction was greater in the exercise than control condition (−17 vs. −4 %, respectively; condition by time interaction *P* = 0.05).

Peak thrombin was 66 % higher in the exercise compared with the control condition (46 to 88 %, ES = 1.37, main effect condition *P* < 0.001), and 67 % higher post-intervention compared with pre-intervention (48 to 90 %, ES = 1.39, main effect time *P* < 0.001). The magnitude of change was greater in the exercise than control condition (184 vs. −3 %, respectively; condition by time interaction *P* < 0.001).

Endogenous thrombin potential was 17 % higher in the exercise compared with the control condition (9 to 25 %, ES = 0.75, main effect condition *P* < 0.001), and 23 % higher post-intervention compared with pre-intervention (15 to 32 %, ES = 0.99, main effect time *P* < 0.001). The magnitude of increase was greater in the exercise than control condition (47 vs. 2 % respectively; condition by time interaction *P* < 0.001).

### Day 2: fasting thrombin generation and plasma concentrations

Fasting plasma thrombin generation parameters and TAG, glucose and insulin concentrations are displayed in Table 1. Linear mixed models revealed no significant differences in fasting plasma thrombin generation between the control and exercise condition for lag time (main effect condition *P* = 0.87), peak thrombin (main effect condition *P* = 0.93) or endogenous thrombin potential (main effect condition *P* = 0.46). Fasting plasma TAG concentration was 13 % lower in the exercise compared with the control condition (ES = 0.46, main effect condition *P* = 0.01). There was no significant difference in fasting plasma glucose (main effect condition *P* = 0.93) or insulin (main effect condition *P* = 0.75) concentration between the control and exercise conditions. No differences were seen between the control and exercise conditions for HOMA-IR [1.29 (0.50) vs. 1.25 (0.47), respectively; 95 % CI −0.26 to 0.18, main effect condition *P* = 0.68] or the insulin sensitivity index [6.65 (2.20) vs. 6.80 (2.55), respectively; 95 % CI −1.07 to 1.36, main effect condition *P* = 0.80].

**Table 1:** Fasting and time-averaged total area under the postprandial concentration vs. time curve in the control and exercise conditions on day 2

	Control	Exercise	Control vs. exercise 95 % CI^a^	Effect size
Time lag				
Fasting (min)	4.29 (3.75–4.91)	4.33 (3.78–4.96)	−11 to 14	0.04
TAUC (min)	4.12 (3.68–4.63)	4.18 (3.72–4.68)	−8 to 12	0.06
Peak thrombin concentration				
Fasting (nM)	73.5 (55.4–97.3)	74.4 (56.1–98.6)	−26 to 39	0.03
TAUC (nM)	89.5 (76.4–104.9)	80.3 (68.5–94.1)	−25 to 7	0.36
Endogenous thrombin potential				
Fasting (nM min^−1^)	1558 (1360–1785)	1475 (1288–1690)	−19 to 11	0.23
TAUC (nM min^−1^)	1708 (1571–1857)	1628 (1497–1770)	−12 to 3	0.32
Triacylglycerol				
Fasting (mmol L^−1^)	0.96 (0.81–1.13)	0.83 (0.71–0.99)	−21 to −4^b^	0.46
TAUC (mmol L^−1^)	1.28 (1.05–1.55)	1.13 (0.93–1.37)	−20 to −2^b^	0.34
Glucose				
Fasting (mmol L^−1^)	5.53 (5.34–5.72)	5.53 (5.35–5.73)	−3 to 3	0.02
TAUC (mmol L^−1^)	5.69 (5.41–5.98)	5.81 (5.53–6.10)	−3 to 7	0.19
Insulin				
Fasting (pmol L^−1^)	28.9 (23.2–36.1)	28.3 (22.6–35.3)	−16 to 14	0.05
TAUC (pmol L^−1^)	156 (131–185)	151 (127–180)	−18 to 14	0.11

Values are geometric mean (95 % confidence interval) for *n* = 16. Statistical analyses are based on natural log transformed data. Comparisons were made using linear mixed models

*TAUC* time-averaged total area under the concentration vs. time curve

^a^95 % confidence interval for the ratio of geometric means

^b^Significant difference between exercise and control conditions (*P* < 0.05)

### Day 2: postprandial thrombin generation and plasma concentrations

No significant differences were seen between the control and exercise conditions for any measure of thrombin generation in the postprandial period (main effect condition *P* ≥ 0.14; condition by time interaction *P* ≥ 0.73). Linear mixed models revealed a significant main effect of time for lag time (*P* = 0.01) and endogenous thrombin potential (*P* = 0.02), but not peak thrombin (*P* = 0.09). Specifically, lag time was lower at 8 h compared with 0 and 2 h (~11 %; *P* ≤ 0.01), and endogenous thrombin potential was elevated at 2, 5 and 8 h compared with 0 h (~11 %; *P* ≤ 0.01). No significant difference in TAUC responses was identified between the control and exercise conditions for any measure of thrombin generation (main effect condition: lag time *P* = 0.79; peak thrombin *P* = 0.21; endogenous thrombin potential *P* = 0.22) (Table 1).

**Fig. 3:**
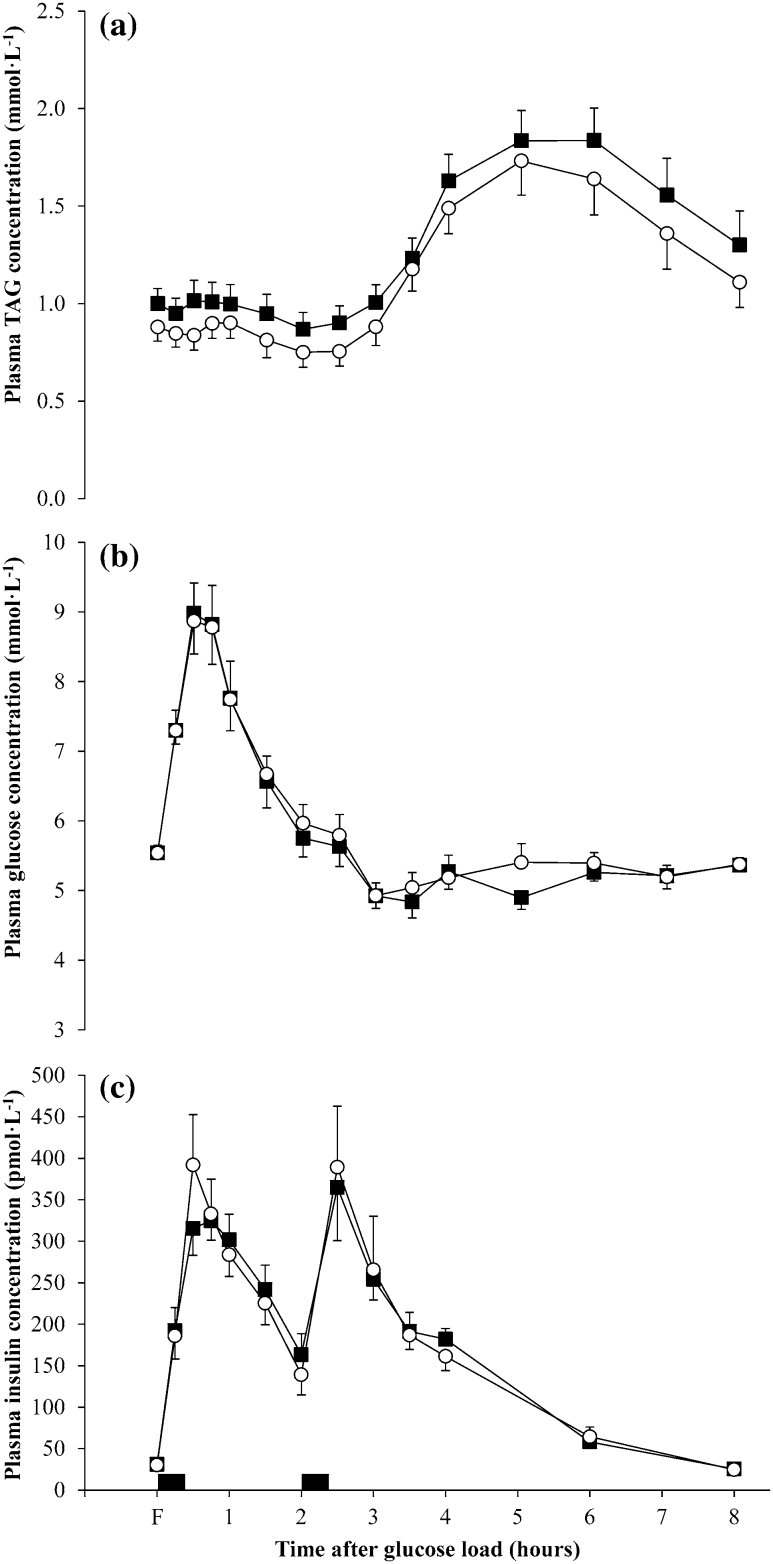
Fasting (F) and postprandial plasma concentrations in the control (*closed square*) and exercise (*open circle*) conditions (*n* = 16): **a** triacylglycerol (TAG); **b** glucose; **c** insulin. Values are mean (SEM). *Black rectangles* denote consumption of a glucose load and standardised meal at 08:00 and 10:00, respectively. There was a significant main effect of condition for TAG (*P* < 0.001)

Plasma TAG, glucose and insulin responses over the postprandial period for the experimental conditions are shown in Fig. 3. Linear mixed models identified differences in postprandial plasma TAG concentrations over time between conditions (main effect condition *P* < 0.001; main effect time *P* < 0.001; condition by time interaction *P* = 0.99). Mean TAG concentration was 12 % lower in the exercise compared with the control condition (−15 to −9 %, ES = 0.29, main effect condition *P* < 0.001). The TAG TAUC was 11 % lower in the exercise compared with the control condition (ES = 0.34, main effect condition *P* = 0.02) (Table 1).

Linear mixed models revealed no differences in postprandial plasma glucose concentration between the conditions (main effect condition *P* = 0.28; main effect time *P* < 0.001; condition by time interaction *P* = 1.00). No significant difference was observed in glucose TAUC between the conditions (main effect condition *P* = 0.38) (Table 1).

Postprandial plasma insulin concentration did not differ significantly between the conditions (main effect condition *P* = 0.39; main effect time *P* < 0.001; condition by time interaction *P* = 0.99). The insulin TAUC was similar between the control and exercise conditions (main effect condition *P* = 0.69) (Table 1).

## Discussion

The primary finding from the present study was that a single session of high-intensity interval rowing increased plasma thrombin generation immediately after exercise in healthy, recreationally active men. However, no differences in postprandial plasma thrombin generation were seen the next day 16–24 h after exercise, but a single bout of interval rowing exercise resulted in a small reduction in postprandial TAG concentrations the next day.

The shortening of lag time immediately after the rowing intervals supports previous studies reporting a shortening of plasma clotting times (e.g., aPTT) after a single session of exercise (Gunga et al. 2002; Hilberg et al. 2002, 2003b; Menzel and Hilberg 2011). However, plasma clot formation occurs before the majority (~95 %) of thrombin is generated and, therefore, thrombin generating processes that take place after clotting has occurred are not reflected in the clotting time (Hemker and Béguin 1995). The substantial increase in the concentration of peak thrombin immediately after the high-intensity rowing intervals appears to be a novel finding. Indirect evidence of increased thrombin generation can be provided by studies reporting post-exercise increases in surrogate markers such as F1 + 2 and TAT (Hilberg et al. 2003a; Weiss et al. 2002); however, this finding is not universal (Eriksson-Berg et al. 2002), but appears more pronounced at higher exercise intensities (Cadroy et al. 2002; Menzel and Hilberg 2011; Weiss et al. 1998) such as the exercise is in the present study.

In contrast to the findings of the present study, previous exercise studies did not observe an increase in endogenous thrombin potential immediately after exercise (Hilberg et al. 2002, 2003a, b). This discrepancy may reflect differences in exercise intensity and duration, but it is more likely due to methodological differences in the measurement of thrombin generation with previous studies using a chromogenic, rather than a fluorigenic, substrate and adjusting for α_2_-macroglobulin-bound thrombin (Hilberg et al. 2002, 2003a, b). Nevertheless, endogenous thrombin potential represents the most robust measure of thrombin generation (Al Dieri et al. 2012), and the findings of the present study suggest that a single session of high-intensity interval rowing provokes a transient prothrombotic tendency.

The present study provides a potential mechanism for the transient increase in risk of cardiovascular complications during and immediately after acute high-intensity exercise. This adds to the debate concerning the potential safety of high-intensity exercise. Although it is well-established that high-intensity exercise induces a transient hypercoaguable state (Posthuma et al. 2015), the risk of acute exercise-induced cardiovascular events is low (Albert et al. 2000; Rognmo et al. 2012). Furthermore, regular exercise training improves markers of coagulation status (Gram et al. 2015; Hilberg et al. 2013), and there is no evidence that the reported health benefits of high-intensity exercise (Currie et al. 2013; Hood et al. 2011; Little et al. 2011; Trombold et al. 2013) are outweighed by the risks (Thompson et al. 2007).

The high-intensity rowing session demonstrated efficacy in reducing postprandial TAG concentrations 16–24 h post-exercise, but elicited no significant or meaningful change in postprandial thrombin generation. While the exact time course of elevated blood coagulation potential in the hours after acute exercise is not known, deleterious perturbations in aPTT, F1 + 2 and TAT appear to subside 3–6 h after strenuous exercise lasting 1 h in duration (Weiss et al. 2002). Previous studies have demonstrated that meal consumption stimulates a transient hypercoaguable state, primarily supported by the activation of factor VII (Gill et al. 2001; Silva et al. 2003), which appears to be positively associated with postprandial TAG concentrations (Silva et al. 2003). In the present study, the small increase in endogenous thrombin potential during the postprandial period in both conditions suggests meal consumption may translate to increased thrombin generation. However, postprandial endogenous thrombin potential was not different between the conditions, suggesting that the exercise-induced changes in postprandial TAG concentrations are independent of changes in endogenous thrombin potential.

Several recent studies have reported reductions in postprandial TAG concentrations after intermittent high-intensity running and cycling (Ferreira et al. 2011; Freese et al. 2011; Gabriel et al. 2012, 2013; Tan et al. 2014; Trombold et al. 2013), although others have demonstrated no change in the postprandial lipaemic response to high-intensity exercise (Allen et al. 2014; Tan et al. 2013). The potential for high-intensity interval rowing to reduce postprandial lipaemia is encouraging considering elevated postprandial TAG concentrations are a strong independent risk factor for cardiovascular disease (Nordestgaard et al. 2007). Furthermore, lack of time and enjoyment are commonly cited barriers to exercise participation (Trost et al. 2002); therefore, high-intensity exercise may offer a time-efficient and for some a more enjoyable strategy to induce metabolic health benefits.

A limitation of the present study is that no markers of fibrinolysis were measured. Several studies have reported that the transient increase in blood coagulation after exercise is matched or even exceeded by augmented fibrinolysis (Hilberg et al. 2003b; Weiss et al. 1998). A further limitation concerns the recruitment of healthy, recreationally active men. Additional research should be conducted to examine if similar responses are observed in patient populations or those who are unaccustomed to high-intensity exercise. However, despite these limitations, this study has provided a meaningful and important insight into the effects of acute high-intensity interval rowing on thrombin generation using a novel assay which is not available currently in the literature.

In conclusion, acute high-intensity interval rowing provokes a transient elevation in plasma thrombin generation immediately after exercise in healthy, recreationally active men. However, postprandial thrombin generation parameters were not different the next day, 16–24 h after exercise, despite a reduction in postprandial plasma TAG concentrations.

## Abbreviations

aPTT: Activated partial thromboplastin time
ES: Effect size
F1 + 2: Prothrombin activation fragment 1 + 2
RPE: Rating of perceived exertion
TAG: Triacylglycerol
TAT: Thrombin-antithrombin complex
TAUC: Total area under the curve
$$\dot{V}$$O_2_: Oxygen uptake
